# PReS-FINAL-1003: Intrinsic cd4 and cd8 effector t cell resistance to suppression in the synovial fluid of juvenile idiopathic arthritis patients

**DOI:** 10.1186/1546-0096-11-S2-P1

**Published:** 2013-12-05

**Authors:** A Petrelli, EJ Wehrens, BJ Prakken, F van Wijk

**Affiliations:** 1 Ped. Immunology, Laboratory of Translational Immunology, UMC Utrecht, Utrecht, The Netherlands

## Introduction

Autoimmune diseases are characterized by an imbalance between regulatory T cells (Treg) and effector T cells (Teff) both in terms of number and function. Our group previously showed that Teff from the site of inflammation (i.e. synovial fluid, SF) of patients affected by Juvenile Idiopathic Arthritis (JIA) are resistant to autologous Treg-mediated suppression irrespective of the source of Tregs (SF or peripheral blood(PB)-derived). However, it is still unclear whether *resistance to suppression *is an intrinsic characteristic of SF-derived Teff or it is induced/maintained by local pro-inflammatory antigen presenting cells (APC).

## Objectives

The aim of this study was to elucidate whether T cells from the SF are *intrinsically resistant *to Treg-mediated suppression.

## Methods

A suppression assay of Cell Trace Violet (CtV)-labeled CD4+CD25- and CD8+ sorted Teff from PB and SF of JIA patients was performed by using anti-CD3 mAb (1.5 μg/ml) plus autologous CD3- cells or anti-CD2/CD3/CD28 beads as stimulators. CtV dilution was used to measure T cell proliferation.

## Results

CD4+ and CD8+ T cells from the SF showed enhanced proliferation compared to the PB. When stimulated with beads, Teff from SF were suppressed by Treg from the same site to a lesser extent then PB Teff. When Teff from the SF were stimulated with anti-CD3 mAb plus CD3- cells from the same site, they failed to be suppressed by Treg.

## Conclusion

These data show that despite SF-derived APC have a role in the induction of Teff resistance to suppression, SF-derived Teff become intrinsically resistant to Treg-mediated suppression.

## Disclosure of interest

None declared.

